# A case report of Williams syndrome with main clinical manifestation of hypercalcemia and gastrointestinal bleeding as the main clinical manifestations, and with an accompanying literature review

**DOI:** 10.1002/brb3.3131

**Published:** 2023-06-20

**Authors:** Hong Meng, Yue‐Xin Jia, Hui‐Min Yang, Xin Gao, Cha‐Gan‐Hu Li, Guo‐Yan Xin, Yu‐Min Wang

**Affiliations:** ^1^ Department of Pediatrics Inner Mongolia Autonomous Region Maternal and Child Health Hospital Hohhot China

**Keywords:** chromosomal diseases, gastrointestinal bleeding, hypercalcemia, Williams syndrome

## Abstract

**Background:**

Williams syndrome is an autosomal dominant multisystem disorder caused by a 1.5–1.8 Mb deletion on chromosome 7q11.23. It is characterized by facial deformations, cardiovascular abnormalities, developmental delays, gastrointestinal manifestations, and endocrine disorders.

**Case description:**

A 1‐year‐old child presenting with developmental delays, special facial features, gastrointestinal bleeding, renal calcium deposition, and hypotonia was admitted to the hospital for “hypercalcemia and gastrointestinal bleeding.” Genetic testing showed a deletion mutation in the 7q11.23 region. Currently, the child receiving treatment to promote calcium excretion and rehabilitation training, but hypercalcemia has recurred.

**Conclusion:**

The clinical phenotype of Williams syndrome is complex, and different severities, characterized by developmental delays, facial deformities, cardiovascular abnormalities, gastrointestinal symptoms and endocrine disorders, should be considered in children. The syndrome may require thorough genetic testing for diagnosis and early intervention treatment to improve patient quality of life.

## Graphical abstract

This is a case of Williams syndrome with gastrointestinal bleeding and literature review.

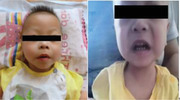

## 1 INTRODUCTION

Williams syndrome (WS) is an autosomal dominant multisystem disorder caused by a 1.5–1.8 Mb deletion on chromosome 7q11.23, which contains approximately 28 genes. Molecular cytogenetic analysis using fluorescence in situ hybridization (FISH), multiple ligation‐dependent probe amplification (MLPA) and other assays can be used to confirm the diagnosis of WS. WS is characterized by facial deformities, cardiovascular abnormalities, developmental delays, gastrointestinal manifestations, and endocrine disorders. Endocrine disorders, such as diabetes mellitus and subclinical hypothyroidism, mainly occur in adults, while hypercalcemia, which occurs with varying frequency and severity, is more common in infants. Mild hypercalcemia occurs in approximately 15% of patients with WS (Barara & Pober, 2010); moderate to severe forms usually occur within the first year of life and resolve naturally by the age of 4 years. Most children with hypercalcemia are asymptomatic at the time of diagnosis, while a few develop symptoms, such as vomiting with secondary weight loss, feeding difficulties, irritability, polyuria, and convulsions. A recent study by Sindhar et al. (2016) showed that a very small number of WS patients (232 patients) had hypercalcemia. Of these patients, only 12 with hypercalcemia required medication to reduce their blood calcium levels. Nephrocalcinosis occurs in 5% of WS patients with hypercalcemia. None of the 11 WS patients from Northern India had hypercalcemia (Gupta et al., 2018).

## 2 CASE REPORT

A boy, aged 1 year and 1 month, was admitted to the Department of Pediatric Medicine of Inner Mongolia Maternal and Child Health Hospital in June 2022 due to “hypercalcemia and gastrointestinal bleeding.” Five months before admission, the patient did not gain weight and was fed with high‐calorie milk powder, but his weight did not increase significantly. Three days before admission, the child had dark red bloody stool, accompanied by poor spirits and decreased milk consumption. The total serum calcium level, measured in the outpatient department of our hospital, was significantly increased by 3.98 mmol/L (2.1–2.8 mmol/L).

### 2.1 Personal history

The child was born at term by caesarean section, with a birth weight of 2460 g, grade III amniotic fluid contamination, and the umbilical cord wrapped around the neck for 1 week. Neonatal Apgar scores were recorded at 1, 7, 5, and 9 min. The child had developmental delays and raised his head at 5 months. At present, he cannot sit or climb alone, and he unconsciously pronounces “baba, mama” sounds. His parents are healthy and nonconsanguineous, deny a family history of hereditary disease, and have no family history of similar hypercalcemia; the child has a healthy 8‐year‐old sister. His parents’ blood calcium and phosphorus levels were normal.

### 2.2 Admission examination

**FIGURE 1:**
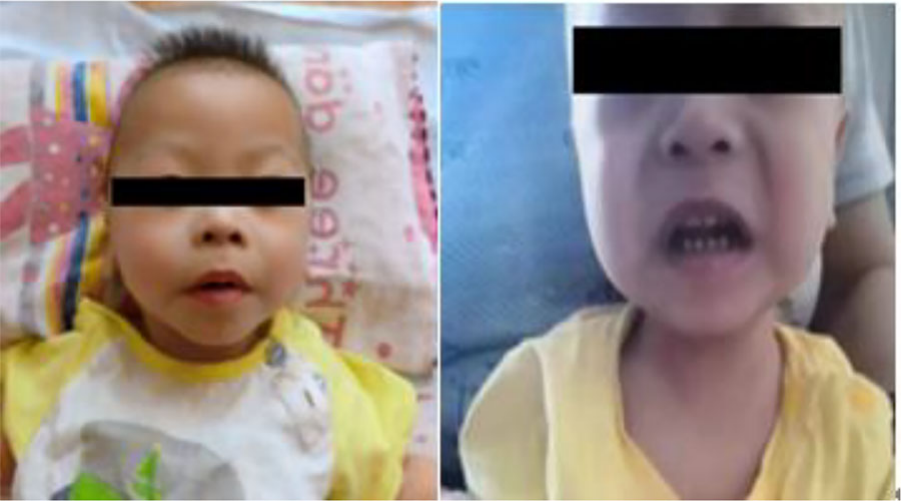
A picture of the patient. The classic facial features of WS include a round forehead, short eye fissures, epicanthus, a full periorbital area, swollen double eyelids, a low and flat nose, full cheeks, thick lips, a medium face length, a large mouth, and small teeth with a sparse arrangement.

The following data were recorded at admission: Body weight: 6.8 kg (less than the third percentile); body length: 70 cm (less than the third percentile); head circumference: 42 cm (less than the third percentile); poor spirits; and a special appearance, including a round forehead, short eye fissures, epicanthus, a full periorbital area, swollen double eyelids, a low and flat nose, full cheeks, thick lips, a medium face length, a large mouth, and small teeth with a sparse arrangement (Figure 1). A grade 2/6 systolic murmur could be heard at the left sternal margin 2–3 intercostal space. Neurological examination revealed normal muscle strength, reduced muscle tone, and no other special features.

### 2.3 Auxiliary examination

Auxiliary tests showed the following (reference values in parentheses): a serum total calcium level of 3.98 mmol/L (2.1–2.8 mmol/L); a magnesium level of 0.94 mmol/L (0.75–1.02 mmol/L); a phosphorus level of 1.00 mmol/L (1.48–2.20 mmol/L); a sodium level of 136.4 mmol/L (134–143 mmol/L); a potassium level of 3.95 mmol/L (4.2–5.9 mmol/L); a parathyroid hormone (PTH) level of 0.39 pmol/L (1.6–6.9 pmol/L); a 25⁃hydroxyvitamin D level > 70 ng/mL (experts recommend a level of at least 30 ng/mL); a urinary calcium/creatinine ratio of 1.35 (normal < 0.2); and a 24‐h urinary calcium level of 7.0 mg/kg (normal < 4 mg/kg). Color echocardiography showed patent foramen ovale (0.24 cm). Urinary color ultrasound showed renal calcium deposition and CT showed multiple patchy slight high‐density shadows in both kidneys. Routine blood examination, liver and kidney function tests, myocardial enzymes, peripheral blood smear, blood gas analysis, thyroid function, urine organic acid analysis, blood amino acid analysis, and carnitine analysis showed no abnormalities.

**FIGURE 2:**
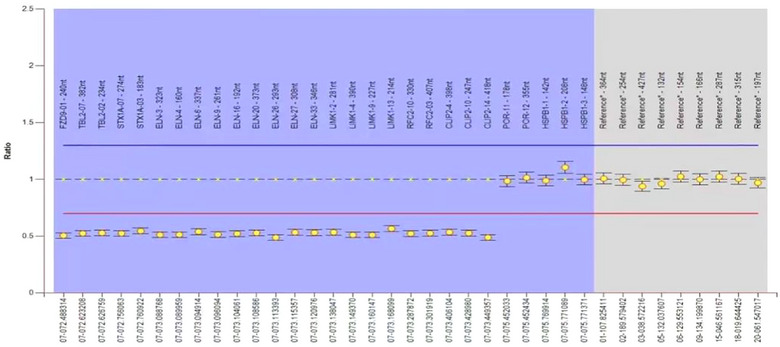
Genetic test results arr 7q11.23 had a 1.78 Mb size variation.

After examining and the patient's family members by using Agilent SurePrintG3HumanGenomeCGHMicroarray (array CGH) 8 × 60 K chip (Agilent Technologies, America), the instructions for comparative genomic hybridization were followed. Luciferin (Cy3/Cy5) was applied to label the DNA of the patient and reference samples, and then microarray hybridization and posthybridization washing were performed. Fluorescence images were obtained by an Agilent chip scanner, the data were read by Feature Extraction Technologies, and standardized processing was performed. Data analysis was performed using Genomic Workbench (Agilent Technologies), and regions of copy number change were found. The position in the genome was determined according to the Human Genome Reference Sequence (GRCH37/HG19), which became available in the UCSC database in February 2009. Gene‐related clinical information via online human Mendelian inheritance (OMIM: 194050) was used to analyze the data. The test results showed (Figure 2) arr 7q11.23 (72,436,289−74,213,788) × 1, 72,436,289−74,213,788, with a variation size of 1.78 Mb. The above variation was not found in the child's parents, so it was considered a novel variation. The diagnosis of WS was confirmed according to the clinical diagnosis score table (Harel et al., 2000) of WS, developed by the American Academy of Pediatrics (AAP), and the genetic testing results.

### 2.4 Treatment and follow‐up

After admission, intravenous fluid replacement and fuemiliuresis were given to promote calcium excretion, temporary water fasting was performed for gastrointestinal bleeding, omeprazole was given for acid inhibition, and symptomatic treatment was given for hemostasis. The patient had a total calcium level of 2.73 mmol/L; a magnesium level of 0.93 mmol/L; a phosphorus level of 1.38 mmol/L; a sodium level of 137 mmol/L; and a potassium level of 5.18 mmol/L. Later, the patient was treated with oral furosemide and potassium citrate. During follow‐up after discharge, the child was treated with oral furosemide tablets (5 mg qd) and potassium citrate granules (0.5 g tid) and vitamin D and calcium intake is avoided. During follow‐up, the child had a recurrence of hypercalcemia, which was improved after receiving corresponding treatment again and has not recurred.

## 3 DISCUSSION

Most cases of WS are sporadic, with little family history. The incidence of WS is 1 in 20,000 live births, and the latest reports show that the incidence can be as high as 1 in 7500 (Brawn & Porter, 2014). The main clinical manifestations of WS are as follows: special features (full periorbital area, low nose bridge, long, small jaw, sparsely arranged teeth, etc.), cardiovascular disease (aortic stenosis, pulmonary stenosis, etc.), abnormal renal function and structure, developmental delays, abnormal personality (overly friendly, anxious, irritable, concentration difficulties, etc.), and gastrointestinal tract anomalies (such as constipation and diverticulitis). WS can also cause ocular diseases, among which strabismus may be the most common manifestation. Hypercalcemia may occur in a small number of children. A total of 10 cases of WS in children with hypercalcemia with complete data reports were collected from the CNKI, Wanfang and PubMed databases from the establishment of the database to October 2022. A total of 11 patients were included in this study. There were no WS cases characterized by “gastrointestinal bleeding” at home or abroad, which is also worthy of attention. Eleven patients had stunted growth, 6 had cardiovascular disease, 6 had irritability, 6 had renal calcinosis, 5 had malnutrition, 4 had low muscle tone, 4 had constipation, 2 had tones, and 2 had increased urination; we included only patients with digestive tract hemorrhage.

The mechanism of hypercalcemia in WS is still unclear. This may be related to the increased absorption and/or decreased clearance of calcium. This increase in intestinal calcium absorption may be due to a higher serum 1,25‐dihydroxyvitamin D level, which suggests vitamin D intoxication and/or a disturbance in the subtle regulation of vitamin D metabolism in the kidney, both of which may contribute to increased calcium absorption. Other patients have normal vitamin D levels but increased sensitivity to vitamin D, which may be related to increased intestinal calcium absorption. Another study suggested that the elevated calcium levels were due to increased calcium release from bone (Lameris et al., 2014). The efficacy of pamidronate and other diphosphonates in reducing calcium levels supports the theory that hypercalcemia in WS patients may be caused by bone calcium release. However, this theory was contradicted by another study that found normal alkaline phosphatase levels in WS patients. Therefore, the mechanisms underlying the development of hypercalcemia are still unclear.

Treatments for hypercalcemia in WS patients include correcting dehydration, increasing renal calcium excretion, and limiting intestinal calcium absorption or bone reabsorption. Treatment in the acute phase includes the discontinuation of vitamin D intake, the administration of a low‐calcium diet, intravenous fluids and furosemide, and careful monitoring of electrolytes to avoid hypokalemia. Adjunctive therapy in the acute phase includes calcitonin, glucocorticoids, and dialysis. Bilal et al. (2018) found that the infusion of phosphonic acid salt may have a good curative effect for WS patients with refractory hypercalcemia. There was a successful use of para metres phosphonic acid sodium treatment in 6 patients. Three patients did not need a calcium diet for a long period of time, but the use of double phosphonic acid salt in the pediatric population still lacks food and drug administration approval.

Hypercalcemia is a clinical emergency. Hypercalcemia crisis occurs when the blood calcium level is higher than 3.5 mmol/L, which can endanger the life of children in severe cases. Patients with hypercalcemia often have no specific clinical manifestations but may present with fatigue, poor appetite, nausea, bone pain, headache, disturbance of consciousness and other manifestations. Urgent calcemia is needed to maintain the stability of the body's internal environment, so it is crucial for the treatment of WS with hypercalcemia. Among these 11 patients, 2 were treated with furosemide and calcium intake restriction, of which 1 had recurrence during follow‐up, and 1 had a good treatment effect. One patient was treated with furosemide and prednisone and another was treated with prednisone and pamidronate, and the effects were good. Furosemide and pamidronate were given for 7 patients; only 1 patient had recurrence, and the rest had good treatment effects. Long‐term treatment was recommended according to the 2001 WS Health Supervision guidelines (Committee on Genetics, 2001), which recommends a low‐calcium diet without vitamin D supplementation and the use of sunscreen to prevent further vitamin D absorption. Long‐term monitoring includes regular monitoring of serum calcium levels at diagnosis, 2 years of age, and 5 years of age, followed by annual monitoring during adolescence and adulthood.

In addition, patients with WS are at greater risk for diverticulitis. WS is caused by the loss of elastin on chromosome 7, one of the main roles of which is to maintain the strength and elasticity of the intestinal wall, and the loss of this protein gene may be the main cause of diverticulitis. A study by the Committee of the German Williams Syndrome Association found that among 128 WS patients aged 18–62.2 years, 14 were diagnosed with sigmoid diverticulitis (Partsch et al., 2005). Another study reported that a 35‐year‐old WS patient developed acute diverticulitis accompanied by severe peritonitis (Bowling Sean & Matthew et al., 2022). Studies have shown that diverticulitis is present in up to one‐third of WS patients, and the youngest reported age for diverticulitis is 9 years old (Ignacio et al., 2012). In our case, gastrointestinal bleeding occurred at the age of 1 year, and the risk of diverticulitis should be monitored. In future follow‐up, we need to pay attention to this symptom, make a timely diagnosis and provide corresponding treatment.

At present, there are still few reports of WS with hypercalcemia, and no cases of gastrointestinal bleeding were found in infants and young children (1 year of age) with WS. WS can present with different degrees of multisystem clinical manifestations. This study describes one patient with WS with manifestations of “hypercalcemia, gastrointestinal bleeding, developmental delays, etc., ” which provides a reference for clinicians to identify this disease. When clinicians encounter children with hypercalcemia, special facial features, developmental delays, gastrointestinal bleeding, constipation, and heart diseases, WS should be considered. Genetic testing techniques such as FISH and MLPA can be used to confirm the diagnosis, and early intervention and rehabilitation training can be provided to improve the quality of life of these patients.

## AUTHOR CONTRIBUTIONS

Hong Meng and Yue‐Xin Jia participated in the clinical practice, including diagnosis, treatment, and consultation. Hui‐Min Yang and Xin Gao contributed to the acquisition of the data. Cha‐Gan‐Hu Li and Guo‐Yan Xin contributed to the analysis of the data. Hong Meng, Yue‐Xin Jia, and Yu‐Min Wang wrote the manuscript. All authors approved the final version of the manuscript.

## CONFLICT OF INTEREST STATEMENT

The authors declare no conflicts of interest.

### PEER REVIEW

The peer review history for this article is available at https://publons.com/publon/10.1002/brb3.3131.

## Data Availability

The datasets generated and/or analyzed during the current study are not publicly available due to the lack of an online platform but are available from the corresponding author upon reasonable request.
